# Crystal structure of 2-bromo­benzoic acid at 120 K: a redetermination

**DOI:** 10.1107/S160053681402087X

**Published:** 2014-09-30

**Authors:** Kornelia Kowalska, Damian Trzybiński, Artur Sikorski

**Affiliations:** a Faculty of Chemistry, University of Gdańsk, W. Stwosza 63, 80-308 Gdańsk, Poland

**Keywords:** crystal structure, 2-bromo­benzoic acid, redetermination, hydrogen bonds, π–π inter­actions

## Abstract

The crystal structure of the title compound, C_7_H_5_BrO_2_, was originally studied using photographic data at room temperature with Cu Kα radiation [Ferguson & Sim (1962 ▶). *Acta Cryst.* **15**, 346–350]. The present study was undertaken at 120 K with a CCD diffractometer using Cu Kα radiation, and resulted in improved geometrical parameters. In the mol­ecule, the carb­oxy group is inclined to the benzene ring by 18.7 (2)° and there is a close intra­molecular Br⋯O contact of 3.009 (3) Å. In the crystal, mol­ecules are linked by pairs of O—H⋯O hydrogen bonds, forming inversion dimers with the classical *R*_2_^2^(8) ring motif for carb­oxy­lic acids. Neighbouring dimers are linked by weak C—H⋯O hydrogen bonds, forming tapes propagating in [1-10]. Adjacent tapes inter­act by slipped parallel π–π inter­actions [inter-centroid distance = 3.991 (2), inter­planar distance = 3.509 (2) Å, slippage = 1.900 Å] to form columns approximately along the *b*-axis direction. Neighbouring columns inter­act dispersively, forming a three-dimensional framework structure.

## Related literature   

For the original report of the unit-cell dimensions, space group and structure of the title compound, see: Ferguson & Sim (1962 ▶). For uses of the title compound in organic synthesis, see: Evano *et al.* (2008 ▶); Wolf *et al.* (2006 ▶), and for its physicochemical properties, see: Govindarajan *et al.* (2011 ▶); Sabbah & Aguilar (1996 ▶); Swaminathan *et al.* (2009 ▶). For related structures involving the title compound, see: Das *et al.* (2012 ▶); Wales *et al.* (2012 ▶). For reports on Br⋯O inter­actions, see: Jones & Lozano (2004 ▶); Saeed *et al.* (2013 ▶); Singh *et al.* (2009 ▶).
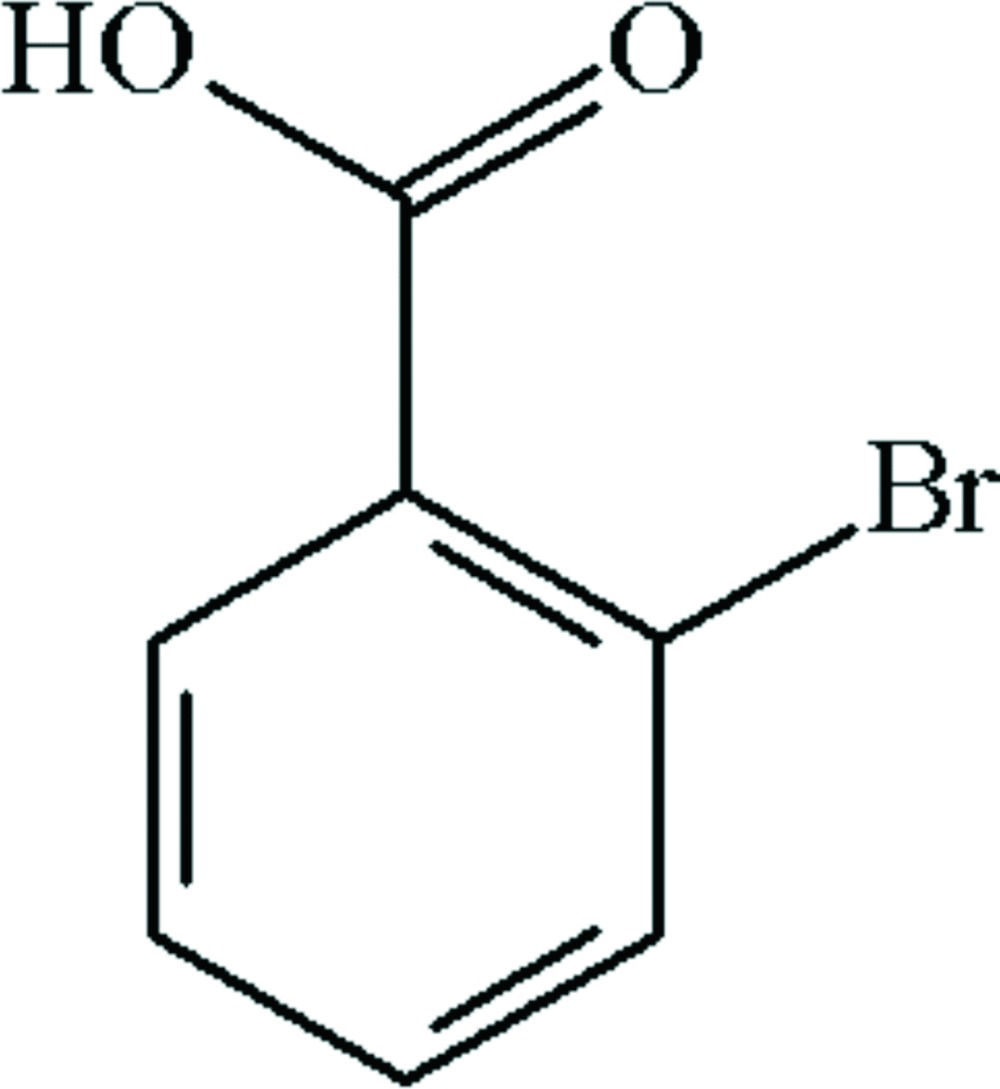


## Experimental   

### Crystal data  

C_7_H_5_BrO_2_
*M* *_r_* = 201.01
Monoclinic, 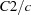

*a* = 14.7955 (4) Å
*b* = 3.99062 (15) Å
*c* = 22.9240 (8) Å
β = 96.906 (3)°
*V* = 1343.69 (8) Å^3^
*Z* = 8
Cu *K*α radiation
μ = 7.76 mm^−1^
*T* = 120 K
0.55 × 0.35 × 0.28 mm

### Data collection  

Oxford Diffraction Gemini R Ultra Ruby CCD diffractometer
Absorption correction: multi-scan (*CrysAlis RED*; Oxford Diffraction, 2008 ▶) *T*_min_ = 0.722, *T*_max_ = 0.991
10883 measured reflections
1201 independent reflections
1172 reflections with *I* > 2σ(*I*)
*R*_int_ = 0.067

### Refinement  

*R*[*F* ^2^ > 2σ(*F* ^2^)] = 0.035
*wR*(*F* ^2^) = 0.091
*S* = 1.18
1201 reflections
95 parameters
1 restraint
H atoms treated by a mixture of independent and constrained refinement
Δρ_max_ = 0.82 e Å^−3^
Δρ_min_ = −0.52 e Å^−3^

### 

Data collection: *CrysAlis CCD* (Oxford Diffraction, 2008 ▶); cell refinement: *CrysAlis CCD*; data reduction: *CrysAlis RED* (Oxford Diffraction, 2008 ▶); program(s) used to solve structure: *SHELXS97* (Sheldrick, 2008 ▶); program(s) used to refine structure: *SHELXL97* (Sheldrick, 2008 ▶); molecular graphics: *ORTEPII* (Burnett & Johnson, 1976 ▶); software used to prepare material for publication: *SHELXL97* and *PLATON* (Spek, 2009 ▶).

## Supplementary Material

Crystal structure: contains datablock(s) global, I. DOI: 10.1107/S160053681402087X/su2783sup1.cif

Structure factors: contains datablock(s) I. DOI: 10.1107/S160053681402087X/su2783Isup2.hkl

Supporting information file. DOI: 10.1107/S160053681402087X/su2783Isup3.cml

**Fig. 1.**
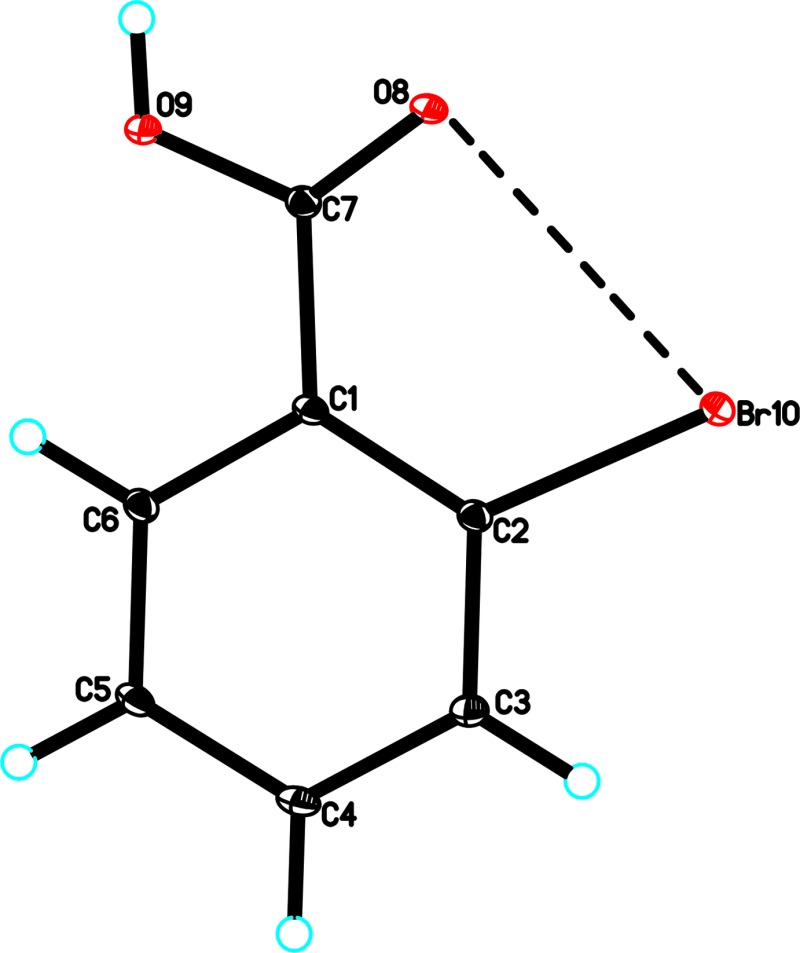
The molecular structure of the title molecule, with atom labeling. Displacement ellipsoids are drawn at the 25% probability level. The short intramolecular Br···O contact [3.009 (3) Å] is shown as a dashed line.

. DOI: 10.1107/S160053681402087X/su2783fig1.tif
The mol­ecular structure of the title mol­ecule, with atom labeling. Displacement ellipsoids are drawn at the 25% probability level. The short intra­molecular Br⋯O contact [3.009 (3) Å] is shown as a dashed line.

**Fig. 2.**
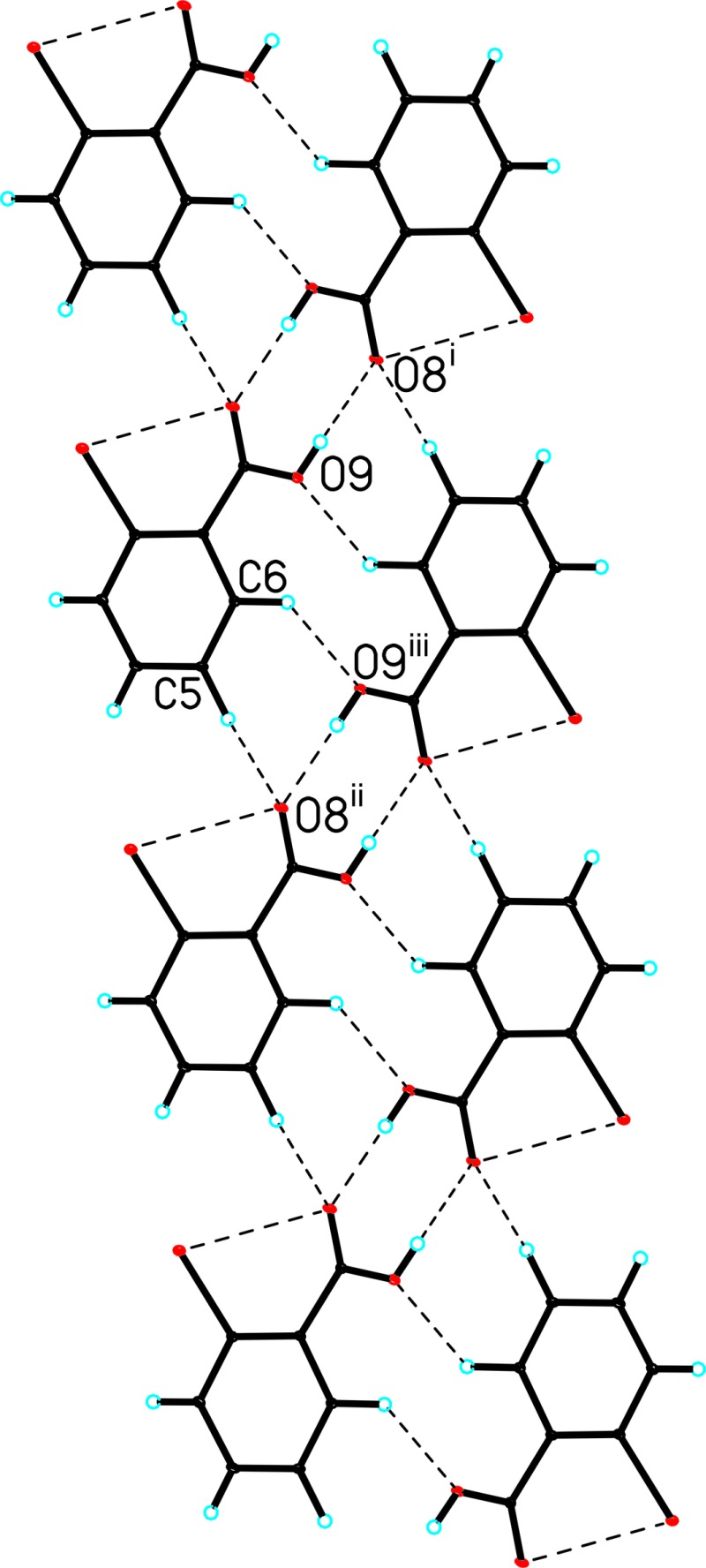
A partial view perpendicular to the ac plane of the crystal packing of the title compound. The O–H···O and C–H···O hydrogen bonds are represented by dashed lines [see Table 1 for details; symmetry codes: (i) –x+1, –y+1, –z+1; (ii) x–1/2, y+1/2, z; (iii) –x+1/2, –y + 3/2, –z+1].

**Fig. 3.**
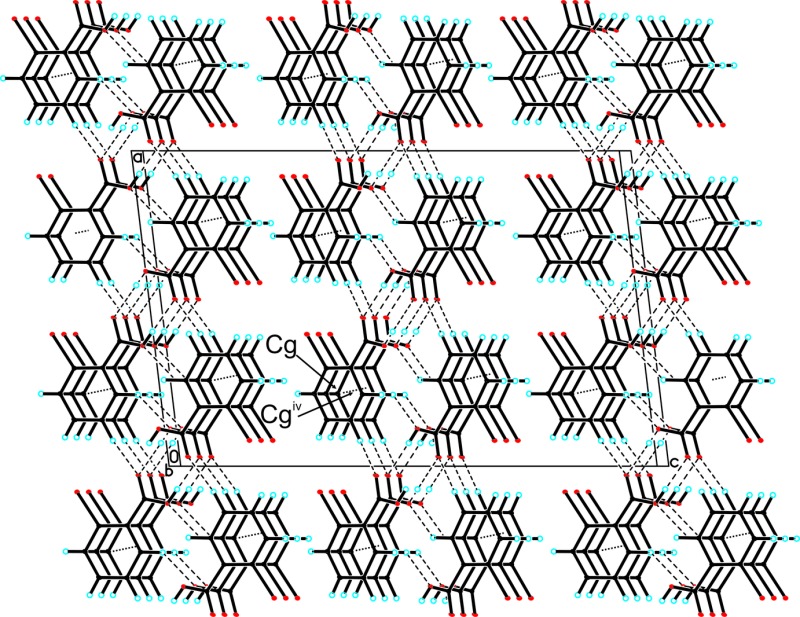
A view along the b axis of the crystal packing of the title compound. The π–π interactions are represented by dashed lines [symmetry code: (iv) x, y+1, z].

Click here for additional data file.ac x y z x y z x y z . DOI: 10.1107/S160053681402087X/su2783fig2.tif
A partial view perpendicular to the *ac* plane of the crystal packing of the title compound. The O–H⋯O and C–H⋯O hydrogen bonds are represented by dashed lines [see Table 1 for details; symmetry codes: (i) –*x* + 1, –*y* + 1, –*z* + 1; (ii) *x*–1/2, *y* + 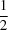, *z*; (iii) –*x* + 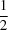, –*y* + 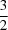, –*z* + 1].

b x y z . DOI: 10.1107/S160053681402087X/su2783fig3.tif
A view along the *b* axis of the crystal packing of the title compound. The π–π inter­actions are represented by dashed lines [symmetry code: (iv) *x*, *y* + 1, *z*].

CCDC reference: 1024798

Additional supporting information:  crystallographic information; 3D view; checkCIF report

## Figures and Tables

**Table 1:** Hydrogen-bond geometry (Å, °)

*D*—H⋯*A*	*D*—H	H⋯*A*	*D*⋯*A*	*D*—H⋯*A*
O9—H9⋯O8^i^	0.81 (3)	1.84 (3)	2.643 (3)	177 (5)
C5—H5⋯O8^ii^	0.93	2.65	3.514 (3)	153
C6—H6⋯O9^iii^	0.93	2.64	3.417 (3)	141

Symmetry codes: (i) 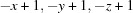; (ii) 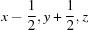; (iii) 
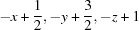
.

## supplementary crystallographic information

### S1. Comment

2-Bromobenzoic acid is a reagent widely used in organic synthesis, for example in cross-coupling reactions (Evano *et al.*, 2008; Wolf *et al.*, 2006). The physicochemical properties of title compound, such as thermodynamic (Sabbah & Aguilar, 1996) and spectroscopic (Govindarajan *et al.*, 2011; Swaminathan *et al.*, 2009) properties, were studied in literature. In 1962, Ferguson and Sim (Ferguson & Sim, 1962) determined the crystal structure of the title compound (*a* = 14.82 Å, *b* = 4.10 Å, *c* = 25.90 Å, *β* = 118.26°, *V* = 1386.2 Å^3^, *R* = 13.20 %), using photographic data at room temperature. Redetermination of the crystal structure of 2-bromobenzoic acid at 120 K shows, that the unit cell dimensions (see: *Experimental section*) differs from those reported previously.

The bond lengths and angles characterizing the geometry of molecule of the title compound (Fig. 1) are similar to those found in other structures containing 2-bromobenzoic acid (Das *et al.*, 2012; Wales *et al.*, 2012). The benzene ring makes an angle of 18.7 (2) ° with the mean plane of the carboxy group. There is also a close intramolecular Br10···O8 contact [3.009 (3) Å; as shown in Fig. 1].

In the crystal, molecules are linked into inversion R_2_^2^(8) dimers by pairs of O9–H9···O8^i^ hydrogen bonds (Table 1 and Fig. 2). Neighbouring dimers are linked by C5–H5···O8^ii^ and C6–H6···O9^iii^ interactions to produce tapes along [1 -1 0] (Table 1 and Fig. 2). Adjacent tapes interact by weak π–π interactions [*Cg*···*Cg*^iv^ = 3.991 (2) Å; Cg is the centroid of the benzene ring C1-C6; interplanar distances = 3.509 (2) Å; slippage 1.900 Å; symmetry code: (iv) x, y-1, z] to form stacked columns approximately along the *b*-axis (Fig. 3). The neighbouring columns interact dispersively to form a three-dimensional framework structure (Fig. 3).

### S2. Experimental

The 2-bromobenzoic acid was purchased from Sigma Aldrich and used without further purification. The single crystals suitable for X-ray investigations were grown by means of slow evaporation of a mixture of ethanol and water (1:1; v:v) solution (m.p. 422.6).

### S3. Refinement

The OH H-atom was located in a difference Fourier map and refined with a distance restraint: O-H = 0.82 (2) Å. The C-bound H atoms were positioned geometrically and constrained to ride on their parent atoms: C–H = 0.93 Å with *U*_iso_(H) = 1.2*U*_eq_(C).

### Crystal data

**Table:**

C_7_H_5_BrO_2_	*F*(000) = 784
*M**_r_* = 201.01	*D*_x_ = 1.987 Mg m^−^^3^
Monoclinic, *C*2/*c*	Melting point: 422.6 K
Hall symbol: -C 2yc	Cu *K*α radiation, λ = 1.54184 Å
*a* = 14.7955 (4) Å	Cell parameters from 10883 reflections
*b* = 3.99062 (15) Å	θ = 3.9–67.3°
*c* = 22.9240 (8) Å	µ = 7.76 mm^−^^1^
β = 96.906 (3)°	*T* = 120 K
*V* = 1343.69 (8) Å^3^	Block, white
*Z* = 8	0.55 × 0.35 × 0.28 mm

### Data collection

**Table:**

Oxford Diffraction Gemini R Ultra Ruby CCD diffractometer	1201 independent reflections
Radiation source: fine-focus sealed tube	1172 reflections with *I* > 2σ(*I*)
Graphite monochromator	*R*_int_ = 0.067
Detector resolution: 10.4002 pixels mm^-1^	θ_max_ = 67.3°, θ_min_ = 3.9°
ω scans	*h* = −17→17
Absorption correction: multi-scan (*CrysAlis RED*; Oxford Diffraction, 2008)	*k* = −4→4
*T*_min_ = 0.722, *T*_max_ = 0.991	*l* = −27→27
10883 measured reflections	

### Refinement

**Table:**

Refinement on *F*^2^	Primary atom site location: structure-invariant direct methods
Least-squares matrix: full	Secondary atom site location: difference Fourier map
*R*[*F*^2^ > 2σ(*F*^2^)] = 0.035	Hydrogen site location: inferred from neighbouring sites
*wR*(*F*^2^) = 0.091	H atoms treated by a mixture of independent and constrained refinement
*S* = 1.18	*w* = 1/[σ^2^(*F*_o_^2^) + (0.048*P*)^2^ + 4.6943*P*] where *P* = (*F*_o_^2^ + 2*F*_c_^2^)/3
1201 reflections	(Δ/σ)_max_ < 0.001
95 parameters	Δρ_max_ = 0.82 e Å^−^^3^
1 restraint	Δρ_min_ = −0.52 e Å^−^^3^

### Special details

**Table:**

Geometry. All esds (except the esd in the dihedral angle between two l.s. planes) are estimated using the full covariance matrix. The cell esds are taken into account individually in the estimation of esds in distances, angles and torsion angles; correlations between esds in cell parameters are only used when they are defined by crystal symmetry. An approximate (isotropic) treatment of cell esds is used for estimating esds involving l.s. planes.
Refinement. Refinement of F^2^ against ALL reflections. The weighted R-factor wR and goodness of fit S are based on F^2^, conventional R-factors R are based on F, with F set to zero for negative F^2^. The threshold expression of F^2^ > 2sigma(F^2^) is used only for calculating R-factors(gt) etc. and is not relevant to the choice of reflections for refinement. R-factors based on F^2^ are statistically about twice as large as those based on F, and R- factors based on ALL data will be even larger.

### Fractional atomic coordinates and isotropic or equivalent isotropic displacement parameters (Å^2^)

**Table:**

	*x*	*y*	*z*	*U*_iso_*/*U*_eq_	
C1	0.3119 (2)	0.3168 (9)	0.40413 (14)	0.0222 (7)	
C2	0.3109 (2)	0.1534 (9)	0.34999 (14)	0.0230 (7)	
C3	0.2293 (2)	0.0629 (9)	0.31713 (16)	0.0268 (8)	
H3	0.2296	−0.0469	0.2814	0.032*	
C4	0.1473 (2)	0.1375 (10)	0.33802 (16)	0.0297 (8)	
H4	0.0927	0.0740	0.3164	0.036*	
C5	0.1463 (2)	0.3045 (10)	0.39040 (15)	0.0277 (8)	
H5	0.0912	0.3580	0.4038	0.033*	
C6	0.2278 (2)	0.3931 (10)	0.42318 (15)	0.0265 (8)	
H6	0.2266	0.5057	0.4586	0.032*	
C7	0.3963 (2)	0.4020 (9)	0.44374 (15)	0.0243 (7)	
O8	0.47061 (15)	0.2776 (8)	0.44031 (11)	0.0337 (6)	
O9	0.38191 (16)	0.6217 (8)	0.48460 (11)	0.0315 (6)	
H9	0.427 (2)	0.659 (12)	0.5071 (15)	0.033 (11)*	
Br10	0.41826 (2)	0.04521 (11)	0.315985 (15)	0.03005 (19)	

### Atomic displacement parameters (Å^2^)

**Table:**

	*U*^11^	*U*^22^	*U*^33^	*U*^12^	*U*^13^	*U*^23^
C1	0.0158 (15)	0.0262 (18)	0.0240 (16)	0.0011 (13)	−0.0009 (12)	0.0025 (14)
C2	0.0188 (15)	0.0263 (18)	0.0232 (16)	0.0026 (13)	−0.0001 (12)	0.0022 (14)
C3	0.0231 (18)	0.031 (2)	0.0248 (17)	0.0008 (14)	−0.0028 (14)	−0.0001 (14)
C4	0.0181 (16)	0.036 (2)	0.0326 (18)	−0.0030 (15)	−0.0053 (13)	0.0049 (16)
C5	0.0154 (15)	0.036 (2)	0.0308 (17)	0.0016 (14)	0.0007 (13)	0.0050 (16)
C6	0.0192 (17)	0.037 (2)	0.0225 (16)	0.0034 (15)	0.0012 (13)	0.0033 (15)
C7	0.0209 (17)	0.0309 (19)	0.0207 (16)	−0.0011 (14)	0.0009 (13)	0.0034 (14)
O8	0.0147 (12)	0.0520 (18)	0.0326 (13)	0.0064 (11)	−0.0052 (9)	−0.0121 (12)
O9	0.0183 (12)	0.0472 (17)	0.0277 (13)	0.0030 (12)	−0.0035 (10)	−0.0109 (12)
Br10	0.0190 (2)	0.0420 (3)	0.0286 (3)	0.00389 (14)	0.00077 (16)	−0.00733 (15)

### Geometric parameters (Å, º)

**Table:**

C1—C2	1.400 (5)	C4—H4	0.9300
C1—C6	1.401 (5)	C5—C6	1.388 (5)
C1—C7	1.492 (5)	C5—H5	0.9300
C2—C3	1.392 (5)	C6—H6	0.9300
C2—Br10	1.901 (3)	C7—O8	1.217 (4)
C3—C4	1.389 (5)	C7—O9	1.319 (5)
C3—H3	0.9300	O9—H9	0.803 (19)
C4—C5	1.375 (5)		
			
C2—C1—C6	117.5 (3)	C3—C4—H4	119.8
C2—C1—C7	124.4 (3)	C4—C5—C6	119.8 (3)
C6—C1—C7	118.1 (3)	C4—C5—H5	120.1
C3—C2—C1	121.1 (3)	C6—C5—H5	120.1
C3—C2—Br10	115.6 (3)	C5—C6—C1	121.5 (3)
C1—C2—Br10	123.3 (2)	C5—C6—H6	119.2
C4—C3—C2	119.7 (3)	C1—C6—H6	119.2
C4—C3—H3	120.2	O8—C7—O9	122.8 (3)
C2—C3—H3	120.2	O8—C7—C1	124.3 (3)
C5—C4—C3	120.4 (3)	O9—C7—C1	112.9 (3)
C5—C4—H4	119.8	C7—O9—H9	113 (3)
			
C6—C1—C2—C3	−1.6 (5)	C4—C5—C6—C1	0.1 (6)
C7—C1—C2—C3	175.8 (3)	C2—C1—C6—C5	1.3 (5)
C6—C1—C2—Br10	177.6 (3)	C7—C1—C6—C5	−176.3 (3)
C7—C1—C2—Br10	−4.9 (5)	C2—C1—C7—O8	−17.1 (6)
C1—C2—C3—C4	0.5 (6)	C6—C1—C7—O8	160.4 (4)
Br10—C2—C3—C4	−178.8 (3)	C2—C1—C7—O9	164.8 (3)
C2—C3—C4—C5	1.0 (6)	C6—C1—C7—O9	−17.7 (5)
C3—C4—C5—C6	−1.3 (6)		

### Hydrogen-bond geometry (Å, º)

**Table:**

*D*—H···*A*	*D*—H	H···*A*	*D*···*A*	*D*—H···*A*
O9—H9···O8^i^	0.81 (3)	1.84 (3)	2.643 (3)	177 (5)
C5—H5···O8^ii^	0.93	2.65	3.514 (3)	153
C6—H6···O9^iii^	0.93	2.64	3.417 (3)	141

Symmetry codes: (i) −*x*+1, −*y*+1, −*z*+1; (ii) *x*−1/2, *y*+1/2, *z*; (iii) −*x*+1/2, −*y*+3/2, −*z*+1.
