# Supplementary figures and images for: Crystal structure of 2-bromo­benzoic acid at 120 K: a redetermination
Source: Acta Crystallogr Sect E Struct Rep Online. 2014 Sep 30;70(Pt 10):o1139–40. doi: 10.1107/S160053681402087X (PMC4257184; doi:10.1107/S160053681402087X)

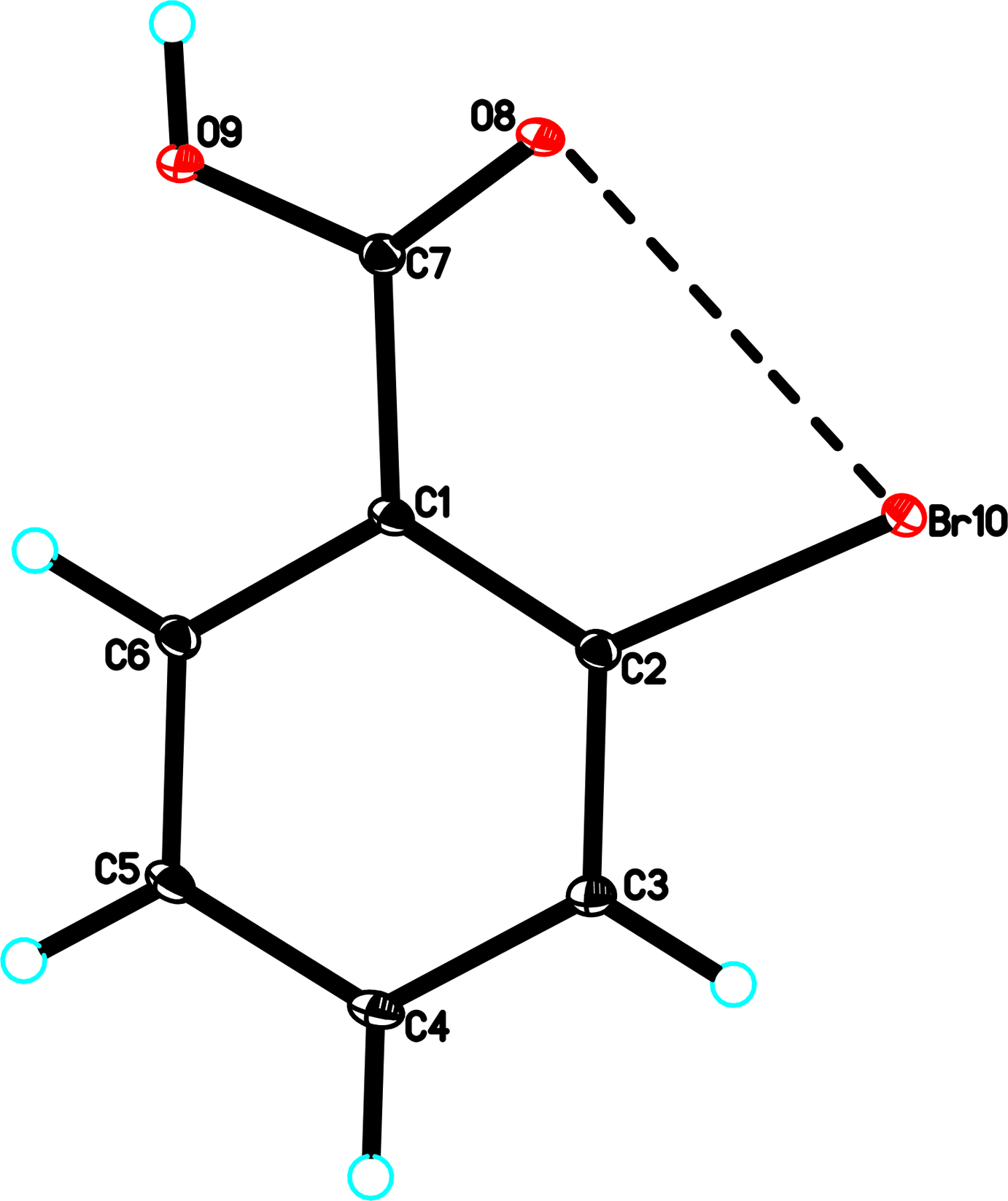

Supplement: Supplementary file 4 [file e-70-o1139-fig1.tif]

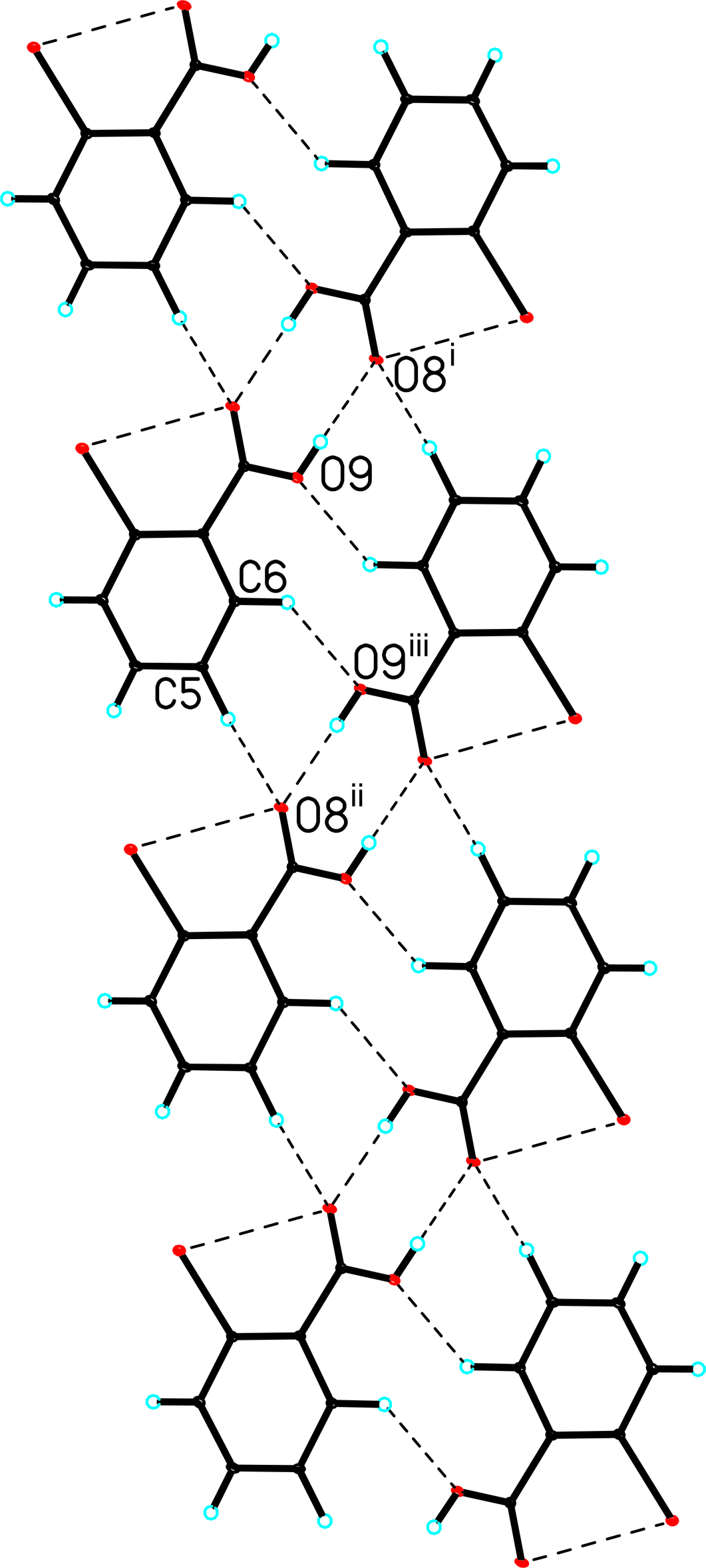

Supplement: Supplementary file 5 [file e-70-o1139-fig2.tif]

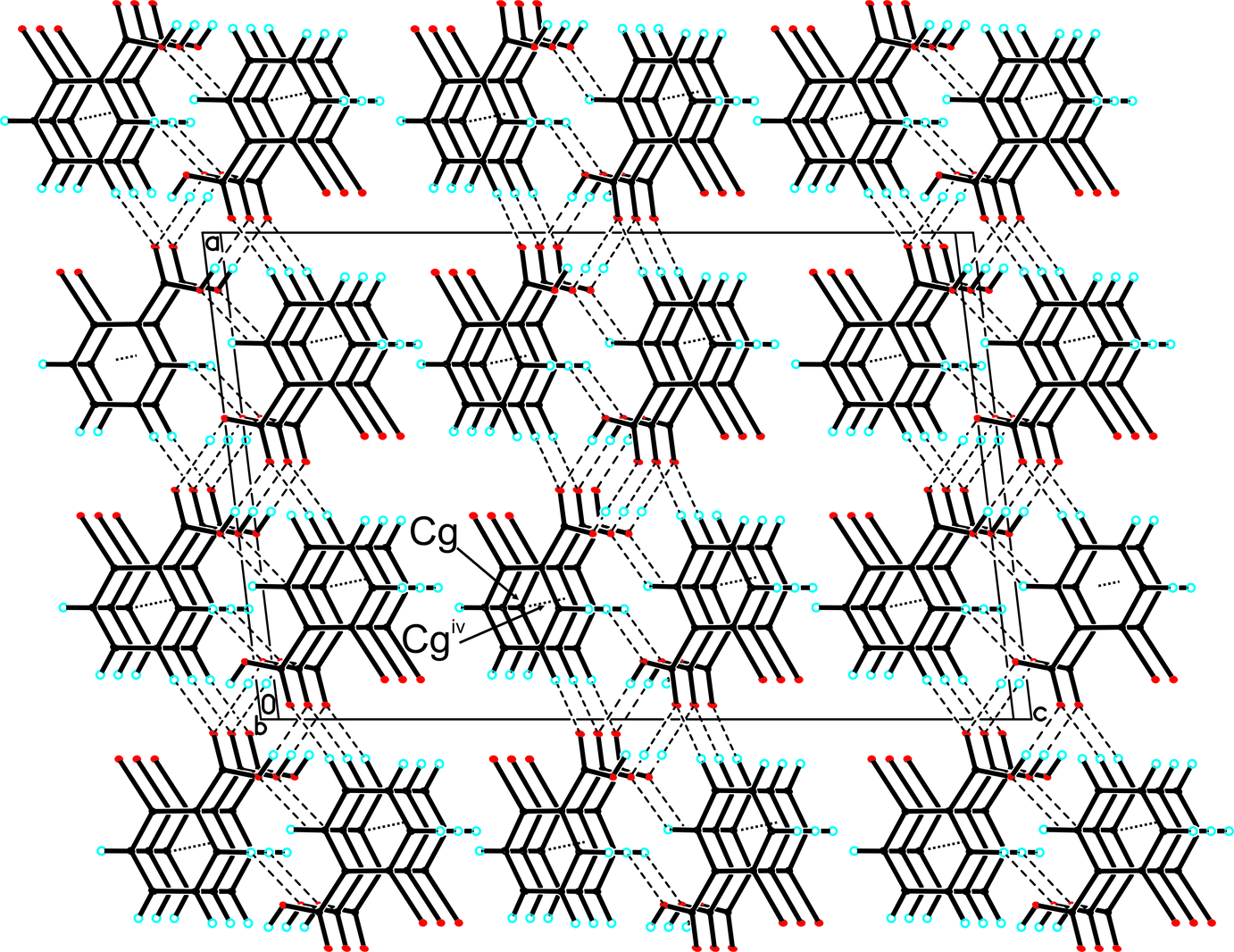

Supplement: Supplementary file 6 [file e-70-o1139-fig3.tif]
